# Optimisation and Characterisation of the Protein Hydrolysate of Scallops (*Argopecten purpuratus*) Visceral By-Products

**DOI:** 10.3390/foods12102003

**Published:** 2023-05-15

**Authors:** Nancy Chasquibol, Billy Francisco Gonzales, Rafael Alarcón, Axel Sotelo, José Carlos Márquez-López, Noelia M. Rodríguez-Martin, María del Carmen Millán-Linares, Francisco Millán, Justo Pedroche

**Affiliations:** 1 Grupo de Investigación en Alimentos Funcionales, Carrera de Ingeniería Industrial, Instituto de Investigación Científica, Universidad de Lima, Av. Javier Prado Este 4600, 15023 Fundo Monterrico Chico, Surco, Lima 15023, Peru; bgonzale@ulima.edu.pe (B.F.G.); ralarcor@ulima.edu.pe (R.A.); alex_94sc@hotmail.com (A.S.); 2 Instituto de la Grasa-Consejo Superior de Investigaciones Científicas, Campus Universidad Pablo de Olavide Ed. 46, Crtra. Sevilla-Utrera km 1, 41013 Sevilla, Spain; jcmarquez@ig.csic.es (J.C.M.-L.); nmrodriguez@ig.csic.es (N.M.R.-M.); mcmillan@ig.csic.es (M.d.C.M.-L.); fmillanr@ig.csic.es (F.M.); j.pedroche@csic.es (J.P.); 3 Department of Medical Biochemistry, Molecular Biology, and Immunology, School of Medicine, University of Seville, 41009 Seville, Spain

**Keywords:** hydrolysis, protein, scallops, by-products, amino acid, enzyme

## Abstract

In this research, scallops (*Argopecten purpuratus*) visceral meal (SVM) and defatted meal (SVMD) were analysed for their proximal composition, protein solubility, and amino acid profile. Hydrolysed proteins isolated from the scallop’s viscera (SPH) were optimised and characterised using response surface methodology with a Box-Behnken design. The effects of three independent variables were examined: temperature (30–70 °C), time (40–80 min), and enzyme concentration (0.1–0.5 AU/g protein) on the degree of hydrolysis (DH %) as a response variable. The optimised protein hydrolysates were analysed for their proximal composition, yield, DH %, protein solubility, amino acid composition, and molecular profile. This research showed that defatted and isolation protein stages are not necessaries to obtain the hydrolysate protein. The conditions of the optimization process were 57 °C, 62 min and 0.38 AU/g protein. The amino acid composition showed a balanced profile since it conforms to the Food and Agriculture Organisation/World Health Organisation recommendations for healthy nutrition. The predominant amino acids were aspartic acid + asparagine, glutamic acid + Glutamate, Glycine, and Arginine. The protein hydrolysates’ yield and DH % were higher than 90% and close to 20%, respectively, with molecular weight between 1–5 kDa. The results indicate that the protein hydrolysates of scallops (*Argopecten purpuratus*) visceral by product optimised and characterised was suitable a lab-scale. Further research is necessary to study the bioactivity properties with biologic activity of these hydrolysates.

## 1. Introduction

*Argopecten purpuratus* is a scallop found in the South Pacific, especially on the coast of Peru and Chile. The leading producers include Peru, Chile, Mexico, and Argentina. Peru is the first producer in Latin America and the fourth worldwide, with 89,872 tm of production [1]. Peru produces scallops, mainly in the regions of Ica, Ancash, and Sechura Bay in Piura, with 75% of the total production [2]. According to Valenzuela et al. [3], scallops are an essential nutritional food because they are low in fat with an exciting amount of omega-3 fatty acids (Alpha Linoleic acid-ALA, Eicosapentaenoic acid-EPA, and Docosahexaenoic acid-DHA), low in carbohydrate and cholesterol with a vital phytosterol content (30%), and a healthy amount of proteins and tryptophan (Trp), as well as vitamin B_12_, and minerals.

However, around 8–35% of the production corresponds to scallop by-products, which could be an excellent opportunity for revaluation [4]. The soft by-products (viscera, mantle, and gills) of *A. purpuratus* have a high protein content (61.36%), 7.5% of fat content, and 30.4% of ω-3 (ALA, EPA, and DHA) fatty acids [5].

Fishing by-products are in demand due to an increasing interest in recovering bioactive compounds, such as protein hydrolysate, bioactive peptides, and amino acids with nutraceutical and bioactive properties. In addition, it is becoming an efficient way to add value to fishing waste [6].

Protein hydrolysates consist of small peptides produced chemically or biologically by hydrolysing the proteins to bioactive peptides that generally contain 2–20 amino acid units [6]. Bioactive peptides exhibit properties such as anti-oxidative, anti-inflammatory, anticancer, neuroprotective, or anti-hypertensive efficacy [7]. Several bioactive compounds, such as linear, cyclic, and conjugated peptides and depsipeptides extracted from bivalve molluscs (scallops, oysters, clams, and other species) have been characterised, and some of them have been approved for use as therapeutic agents and supplements [8]. The molluscs *Tympanotonus fuscatus* var. radula (L.) and *Pachymelania aurita* (M.) demonstrate antioxidant activity as assayed by 2,2-diphenyl-1-picrylhydrazyl (DPPH) method and an Angiotensin-I-converting enzyme (ACE) inhibitory activity with molecular weights (MW) ≤ 3 kDa in the hydrolysate fractions [9]. Another study showed that the hydrolysates fractions (MW < 5 kDa) of oyster *Perna canaliculus* presented anti-hypertensive and antioxidant activities [10]. The fraction hydrolysates of the clam *Tegillarca granosa* with an MW of 0.398 kDa showed antioxidant and anticancer activities, as determined by Chi et al. [11]. The hydrolysates of scallops (*Chlamys farreri*) protein presented low-molecular-mass peptides (10–15 kDa) with linoleic acid peroxidation inhibition and free radical scavenging activities [12].

Two methods have been described to generate protein hydrolysates: chemical and enzymatic hydrolysis; the latter has been reported as the most promising process [13]. Enzymatic hydrolysis produces protein hydrolysates that can be described as fast, safe, and easily controllable [7]. Several factors, such as the type of enzyme, pH, time, temperature, enzyme-substrate ratio, solid-liquid ratio, and enzyme concentration, influence and determine the different biological activities observed because they produce different peptides [14]. Despite multiple investigations with experimental conditions established, the hydrolysis process can be optimised using a response surface methodology (RSM) to reach the maximum DH % employing a design of experiments varying the parameters as temperature, enzyme ratio, time, pH, and others [15,16,17]. RSM is a collection of mathematical-statistical technique useful for designing experiments, developing, and optimizing processes to attain a target response [18].

This study aimed to optimise protein hydrolysis process of *A. purpuratus* by-products using the response surface methodology (RSM) with Box-Behnken design to reach the maximum DH %, and to characterise the protein hydrolysate for, degree of hydrolysis, proximal composition, protein curve solubility, amino acid composition, and molecular weight profile.

## 2. Materials and Methods

### 2.1. Raw Materials

Scallop visceral by-products (SVBP) (*Argopecten purpuratus*) were collected by Gam Corp SA enterprise from the Sechura Bay, Piura Department—Peru. The SVBP were frozen and sent to the Functional Foods Laboratory of the Universidad de Lima–Peru. Frozen SVBP were thawed, washed, and dehydrated by an infrared dryer (IRD D18, Sevvilla, Spain) at 60 °C for 12 h. Then, samples were ground (Grindomix GM200/Restch, Haan, Germany) to obtain the SVBP meal (SVM) and kept in aluminised bags at room temperature for later analysis. The samples of SVM were defatted with hexane for 6 h to obtain defatted SVM (SVMD).

Alcalase (2.4 L) was purchased from Sigma Chemical (St. Louis, MO, USA). All chemical compounds (reagents and solvents) were provided by Sigma Chemical (St. Louis, MO, USA) and Pharmacia Biotech and were of analytical grade.

### 2.2. Experimental Design for Optimisation of the Degree of Hydrolysis

#### 2.2.1. Enzymatic Hydrolysis Reaction

The enzymatic hydrolysis reaction was carried out according to the method described by Millan-Linares et al. [19] with some modifications, using continuous stirring under controlled pH, temperature, and time conditions, using a fermenter-bioreactor (TEC-BIO-FLEX-II, Sao Paulo, Brazil). The scallop viscera meal (SVM) was resuspended in distilled water (10% *w/v*) in a temperature range of 30–70 °C in 100 mL of total volume of reaction. Alcalase enzyme was added in an enzyme/substrate ratio between 0.1–0.5 AU/g protein (pH 8) for 40–80 min with constant stirring at 1000 rpm and maintaining the pH with NaOH (0.1 N). Each sample was heated at 80 °C for 15 min to ensure complete inactivation of remaining enzyme activity. The sample was centrifuged at 10,000 rpm for 15 min. The supernatant of each experimental assay was used to determine the DH% using the Design of the Experiment (DOE).

#### 2.2.2. Design of Experiment (DOE)

To optimise the DH%, the experimental design employed a response surface methodology (MSR) with a Box-Behnken design (BBD) [20], using the Minitab 19 software (Stat-Ease, Inc., Minneapolis, MN, USA). Three independent variables were used: X1—temperature, °C; X2—time, minutes; and X3—enzyme/substrate level, AU/g of protein at three equidistant levels (−1, 0, and +1). The DH % was determined as the response variable (Y). The coded variables are found in Table 1, the range of independent variables was selected according to the Alcalase (2.4 L) technical data sheet and measurement were made in triplicate.

The fifteen experimental trials of the Box-Behnken design are shown in Table 2. The data from each experimental trial were applied according to the MSR through the proposed reduced analysis of variance (ANOVA) models. The experimental data were fitted using the quadratic model (1):

(1) $$Y=\beta_{0}+\sum_{j=1}^{3}\beta_{j}X_{j}+\sum_{j=1}^{3}\beta_{jj}X_{j}^{2}+\sum_{i}\sum_{<j=2}^{3}\beta_{ij}X_{i}X_{j}+e_{i}$$

where Y is the dependent variable. $X_{i}$ and $X_{j}$ are independent variables. $\beta_{0}$, $\beta_{j}$, $\beta_{jj}$, and $\beta_{ij}$ are variable regression coefficients for the intercept, linear quadratic, and interaction effects of the model, respectively. The error is represented by $e_{i}$. The adequacy of the generated mathematical models was determined in terms of their determination coefficients $R^{2}$, $R_{adj}^{2}$ and adequate precision values. The optimisation of the hydrolysis process was determined from the mathematical model selected to determine the conditions for the highest DH%.

#### 2.2.3. Degree of hydrolysis

The degree of hydrolysis was determined in fifteen Box-Behnken experimental design trials (Table 2) for the optimised samples of the scallop viscera meal protein hydrolysates. The DH % was determined by the reaction of the free amino groups with 2,4,6-trinitrobenzenesulfonic acid (TNBS) [21]. An aliquot of 0.25 mL of the hydrolysate was added in a tube with 2 mL of sodium dodecyl sulfate (SDS) (1%) and incubated for 15 min at 80 °C. Then, it was centrifuged, and 0.25 mL of the supernatant was added to tubes containing 2 mL of Na_3_PO_4_ buffer solution (0.2125 M, pH = 8.2). Immediately, 2 mL of 0.025% TNBS was added to each tube and incubated in the dark at 50 °C for 60 min. Then 4.0 mL of HCl (0.1 N) was added. The tubes were cooled, and the absorbance was read on the UV-Vis spectrometer (Shimadzu UV-1280, Kyoto, Japan) at 420 nm. The calibration curve was constructed with L-leucine (0.10–2.5 mM). The %*DH* was calculated using the Equation (2):

(2) $$\%DH=\frac{{AN}_{2}-{AN}_{1}}{N_{pb}}$$

where *AN*_1_ is the amino nitrogen content of the protein substrate before hydrolysis (control) (mEq-NH_2_/g protein), *AN*_2_ is the amino nitrogen content of the protein substrate after hydrolysis (mEq-NH_2_/g protein) and *N_pb_* is the nitrogen content of the peptide bonds in the protein substrate (mEq-NH_2_/g protein).

### 2.3. Protein Hydrolysate

The hydrolysed proteins were obtained from SVM and SVMD, as described in Section 2.2.1. The processes were carried out to the method described by Millan--Linares et al. [19]. SVM and SVMD samples were dissolved in water (10% *w/v*) at 50 °C. Alcalase enzyme was added at an enzyme/substrate ratio of 0.3 AU/g protein (pH 8) for 60 min using a magnetic stirrer (Stuart, Saint Neots, UK). To ensures complete inactivation of enzyme activity, each sample was heated at 80 °C for 15 min. The samples were centrifuged at 10,000 rpm for 15 min. The supernatants from all samples were lyophilised. The lyophilisation process was carried out in the Freeze Mobile 3 model freeze drier equipment (VirTis Co., Gardiner, NY, USA) at −38 °C and a vacuum pressure of 60 atm. Finally, freeze-dried scallop protein hydrolysate (SPH_FD_) and freeze-dried defatted scallop protein hydrolysate (DSPH_FD_) were obtained. The samples were packed until use.

### 2.4. Proximal Composition

The proximal composition (moisture, protein, fat, carbohydrates, and ash) was carried out according to official methods. The moisture content was performed at 110 °C until constant weight (UNE-EN ISO 665:1958). The total protein content was performed by elemental analysis with a nitrogen-to-protein conversion factor of 6.25 in LECO CHNS-932 (St. Joseph, MI, USA). In a muffle furnace, the ash content was determined by incineration at 550 °C for 72 h (UNE 050:1994). The fat content was determined with hexane by the Soxhlet method (UNE-EN ISO 659:1968). The fibre content was measured using the method described by Lee et al. [21]. All measurements were performed in triplicate.

### 2.5. Determination of Amino Acid Composition by High-Performance Liquid Chromatography (HPLC)

The amino acid composition was determined according to the method described by Alaiz et al. [22] with slight modifications for the scallop visceral meal samples with and without fat and the hydrolysed proteins with and without fat. Samples (2–4 mg of protein) were hydrolysed by incubation in 4 mL of HCl (6 N) at 110 °C for 24 h in tubes sealed under nitrogen. After hydrolysis, the samples were dried using a rotary evaporator (Buchi rotavapor R 100 Labortechnic AG, Flawil, Switzerland) and later re-dissolved in sodium borate 1M, sodium azide 0.02%, at pH 9.0. Amino acids were determined in the acid hydrolysate by high-performance liquid chromatography (Acquity Arc, Waters, Milford, MA, USA) after derivatisation with diethyl ethoxymethylenemalonate at 50 °C for 50 min, using D, L-α-aminobutyric acid as internal standard, and a 300 mm × 3.9 mm reversed-phase column (Nova Pack C18 4 µm; Waters, Milford, Massachusetts, USA). A binary gradient system was used with two solvents: (A) 25 mM sodium acetate 0.02% sodium azide (pH 6.0), and (B) acetonitrile. The calibration curves for each amino acid were developed using a mix of the amino acid standards at the same hydrolysis conditions of the samples (Merck, Madrid, Spain), and the resultant peaks were analysed with EMPOWER software (Waters, Santa Clara, CA, USA). Furthermore, tryptophan content was assessed according to the method described by Yust et al. [23]. All measurements were performed in triplicate.

### 2.6. Protein Solubility Curve

The protein solubility curve was determined according to the method described by Paz et al. [24] with some modifications. The visceral scallop meal with and without fat and the hydrolysed proteins with and without fat were dissolved in H_2_O (5% *w/v*), and the pH was adjusted to 2–12 with 1N NaOH or 1N HCl and kept under stirring for 1 h at room temperature. At each pH point (2, 4, 6, 8, 10, and 12), an aliquot was taken in duplicate and centrifuged for 15 min at 10,000 rpm. In the recovered supernatants, the nitrogen content was determined in the LECO CHNS-932 analyser (St. Joseph, MI, USA). The results were expressed as % of the total protein content of the solubilised protein. The supernatant protein was estimated as % nitrogen content × 6.25. All measurements were performed in triplicate.

### 2.7. Molecular Weight Profile by Exclusion Chromatography (SEC)

Molecular weight profiles of fat-free and fat-hydrolysed proteins were estimated using AKTApurifier 10 exclusion chromatography (GE Healthcare Bio-Sciences AB, Uppsala, Sweden, according to Paz et al. [24]. A size separation column was used, molecular: Superose 12 10/300 GL (GE Healthcare) with a separation range between 1–300 kDa Proteins with known molecular weight were used to calibrate the Superose 12 HR 10/30 column (Pharmacia Biotech, Stockholm, Sweden). The following standards were used: dextran blue (2000 kDa) (Pharmacia Biotech), catalase (240 kDa) (Serva, Heidelberg, Germany), bovine serum albumin (67 kDa), ovalbumin (43 kDa), ribonuclease (13.7 kDa), cytochrome C (12.5 kDa) (Pharmacia Biotech), and bacitracin (1.45 kDa) (Sigma Chemical Co, St. Louis, MO, USA.) Line calibration was performed using log molecular weights of control proteins and their elution volumes. Elution was performed with sodium phosphate buffer (0.05 M), sodium chloride (0.2 M), and sodium azide (0.02% *w/v*) with a flow rate of 1 mL/min at pH 7.5. Around 500 μL of the samples with an initial concentration of 5% of the sample (p/v) was used. The recorded elution of proteins was measured at 280 nm absorbance.

### 2.8. Statistical Analysis

The results were expressed as the mean ± standard deviation. All measurements were determined in duplicate or triplicate. The ANOVA was used to analyse the acquired data at a 95% significance level. The Box-Behnken design was evaluated using the response surface methodology with Minitab 19.0 Software (Minitab Inc., State College, Palo Alto, CA, USA).

## 3. Results and Discussions

### 3.1. Proximal Composition of Scallops Visceral Meal

The proximal composition of scallop viscera meal (SVM) and defatted scallop viscera meal (SVMD) are shown in Table 3.

The moisture content of both samples varied between 1.75% and 7.03%. The moisture content of the SVM sample was lower than that reported by Colán-Ramos et al. [5] (11.7%) using the same raw materials and Benítez-Hernández et al. [25] (5.3%) in Catarina scallop viscera meal. It was similar to Lúquez-Pérez and Hleap-Zapata, [26] (1.20–2.8%) in fish viscera meal. The ash content of the SVM (9.54%) and SVMD (9.29%) samples were lower than those reported by Colán-Ramos et al. [5] (11.03%), Lúquez-Pérez and Hleap-Zapata, [26] (11.06–15.19%) but similar to Benítez-Hernández et al. [25] (8.6%).

The fat content was 7% for the SVM sample, similar to Colán-Ramos et al. [5] (7.5%), but lower than the reported by Benítez-Hernández et al. [25] (30%) and fish viscera meal (30%) reported by Lúquez-Pérez and Hleap-Zapata [26]. The results showed that the protein content of the SVM and SVMD samples was 72.23% and 73.33%, respectively. These values were higher than those reported by Benítez-Hernández et al. [25] (57.77%) and Colán-Ramos et al. [5] (61.36%), and Lúquez-Peréz and Hleap-Zapata [26] (40.12–49.62%).

### 3.2. Amino Acid Profile of Scallops Visceral Meal

The amino acid composition of the SVM and SVMD samples is indicated in Table 4. The predominant amino acids were aspartic acid + asparagine (Asp + Asn), glutamic acid + glutamine (Glu + Gln), glycine (Gly), and arginine (Arg). The samples showed similar profiles except for Asp + Asn in the SVM sample (66.57 mg/g) and lower in SVMD (47.74 mg/g). Despite the need for more information on the amino acid profile of *A. purpuratus* and its by-products, it is possible to compare it with other raw materials. Tabakaeva et al. [27] reported that the essential amino acid Trp (7.0–10 mg/g protein) was found in small amounts in some parts of clams (*Anadara broughtonii* and *Mactra chinensis*) such as muscle, mantle, and adductor, values very similar to those found in our samples (0.73% in SVM and 0.71% in SVMD). Xing et al. [28] studied the protein content and the amino acid profile of the viscera of the Chinese scallop (*Patinopecten yessoensis*) and showed that (Phe + Tyr) were essential amino acids with the highest content (8.57%) in organ meats; this is consistent with the amino acid profile of SVMD and SVM (7.22% and 7.88%, respectively). The glycine (12.46%) content of SVM agreed with the reported by Xing et al. [28] (11.93%), but the methionine + cysteine (9.25%) content was higher (3.81%). Methionine and cysteine are sulfur amino acids that are related with key aspect on human health and cellular functions [29]. On the other hand, the amino acid composition of SVM showed a balanced profile because it conforms to the Food and Agriculture Organisation/World Health Organisation (FAO/WHO) recommendations for healthy nutrition. In addition, the sum of amino acids was close to 64%, indicating that the protein content reported in Table 2 would be overvalued data due to the conversion factor (6.25). This could be due to non-protein nitrogenous compounds in the SVM sample, as in other plant species where protein conversion factors lower than 6.25 are recommended.

### 3.3. Protein Solubility Curve of Scallops Visceral Meal

**Figure 1:**
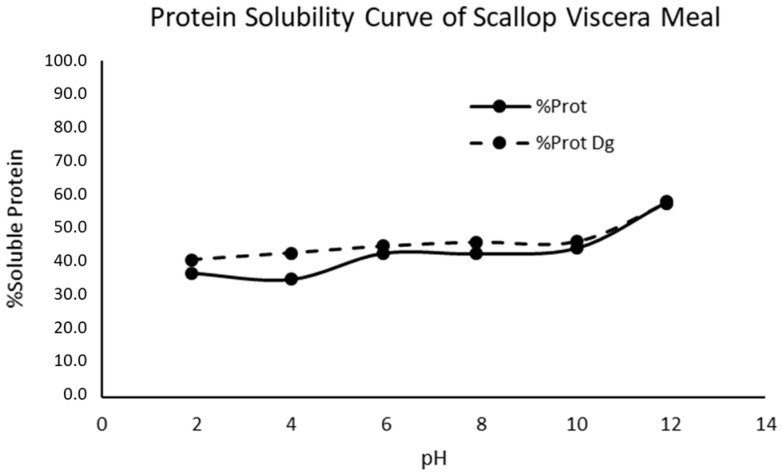
Protein solubility curve of scallop viscera meal. % Prot: Scallop visceral meal (SVM) and % Prot Dg: defatted SVM (SVMD).

The protein solubility curve is shown in Figure 1. The protein solubility for SVMD was slightly higher than SVM in both curves. The highest protein solubility was around 60% at pH 12, and the lowest was around 40% at pH 2. Surasani et al. [30] declared that the maximum solubility of seafood materials was found at pH 2.0–3.0 and pH 11.0–13.0, while the minimum solubility was around pH 5.0–6.0. The curve presented in this study was not the typical bell-shaped curve; that was in contrast with the standard curve presented by several authors, such as Abdollahi and Undeland [31], Mahdabi and Hosseini Shekarabi [32], and Sathe et al. [33], where there were three distinguished zones: acidic and alkali side, and minimum-point solubility side. It was not presented minimum-point solubility or isoelectric point; this makes difficult a purification process over the pH. Therefore, the hydrolysis was performed without the protein isolation process, also considering the high protein percentage of the starting flour (64% by amino acid composition; 72% by elemental analysis using a protein conversion factor of 6.25). On the other hand, any defatting process is necessary as a pre-treatment for enzymatic hydrolysis.

### 3.4. Optimisation of Enzymatic Hydrolysis

The optimal conditions for the enzymatic hydrolysis process of scallop viscera meal were predicted using the response surface methodology with a Box-Behnken design. Table 5 shows the *p*-value of the coefficients related to the following terms: Temperature (°C)—A, Time (min)—B, and Enzyme/substrate ratio (AU/g of protein)—C. All the terms resulted in a *p*-value less than or equal to α = 0.05, so there is an association between the response variable and the terms presented, except for the BC interaction. This would indicate that the Time (min)-B associated with the enzyme/substrate ratio (AU/g of protein)-C would have little significance in explaining the quadratic model. On the other hand, regression statistics, such as the coefficient of determination R^2^ (91.48%), adjusted coefficient of determination R^2^adj (89.29%), and predicted coefficient of determination R^2^pred (84.91%), would indicate an acceptable degree of correlation between the model and the response variable.

**Figure 2:**
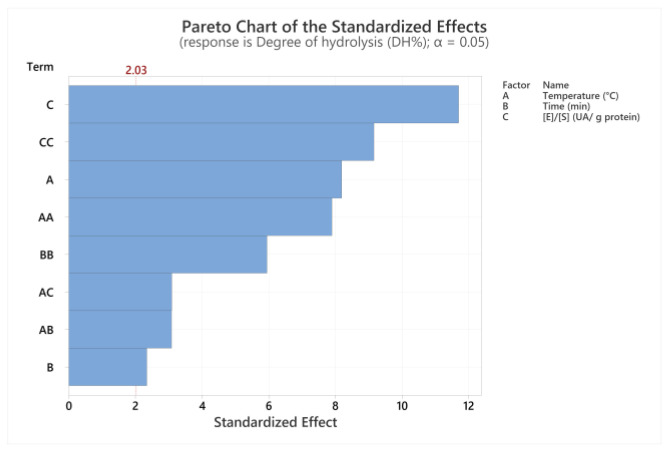
Pareto Chart of the standardized effects.

Table 6 shows the ANOVA of the Box-Behnken design. According to the *p*-value, the quadratic model (0.000) could explain the response variable since it is less than α = 0.05. However, the *p*-value of the BC interaction (Time (min)—B *Enzyme/substrate ratio (AU/g protein)—C) of the quadratic model is more significant than α = 0.05, so this term would have little importance in the model. This could be properly noted in detail in the Pareto chart (Figure 2). Regarding the lack of fit test, the *p*-value was less than α = 0.05 (significant), so the model does not correctly estimate the regression.

The response variable was related to the variables through the regression equation of the second-order quadratic model:

DH (%) = −33.06 + 0.809 Temperature (°C) + 0.595 Time (min) + 85.32 [E]/[S] (UA/g protein) − 0.007860 Temperature (°C) × Temperature (°C) − 0.005920 Time (min) × Time (min) − 91.17 [E]/[S] (UA/g protein) × [E]/[S] (UA/g protein) + 0.002951 Temperature (°C) × Time (min) − 0.2959 Temperature (°C) × [E]/[S] (UA/g protein).(3)

**Figure 3:**
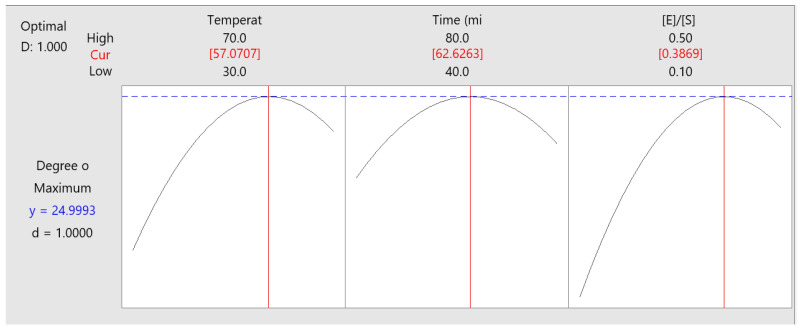
Optimisation of the Box-Behnken design obtained from the Minitab 19 software.

The optimisation of the DH % through the second-order quadratic model is presented in Figure 3. The optimal conditions of the Box-Behnken design were: temperature (57.07 °C), time (62.62 min), enzyme/substrate (0.3869 AU/g protein) with a calculated DH% of 24.99%. This optimisation process was validated in an additional experimental in 2 L of total volume of reaction, where the DH% was 21.4 ± 0.25. On the other hand, DH% calculated was close to the value reported in the central experimental runs (Table 2). Therefore, the freeze-dried hydrolysed proteins of the scallop viscera were obtained at the central point conditions (50 °C, 60 min, and 0.3 AU/g protein) since was needed less energy to get at 50 °C than 57.07 °C, and in order to get 0.3 AU/g protein less enzymatic solution are required to obtain similar DH (%). The optimization process was done only with SVM. Further studies are needed for the optimisation process of the sample SVMD.

**Figure 4:**
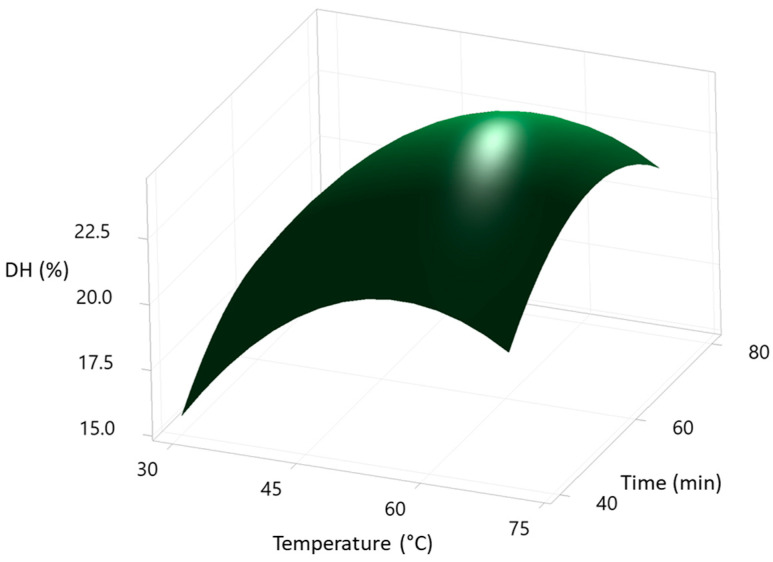
Surface plot of the degree of hydrolysis (DH%) of SVM sample vs. time (min), temperature (°C). Hold Value: [E]/[S] (0.3 UA/g protein).

**Figure 5:**
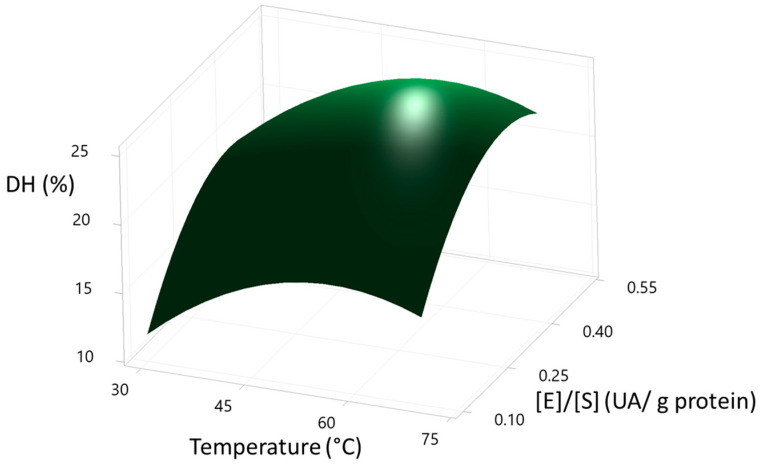
Surface plot of the degree of hydrolysis (DH%) of SVM sample vs. [E]/[S] (UA/g protein), temperature (°C). Hold Value: Time (60 min).

**Figure 6:**
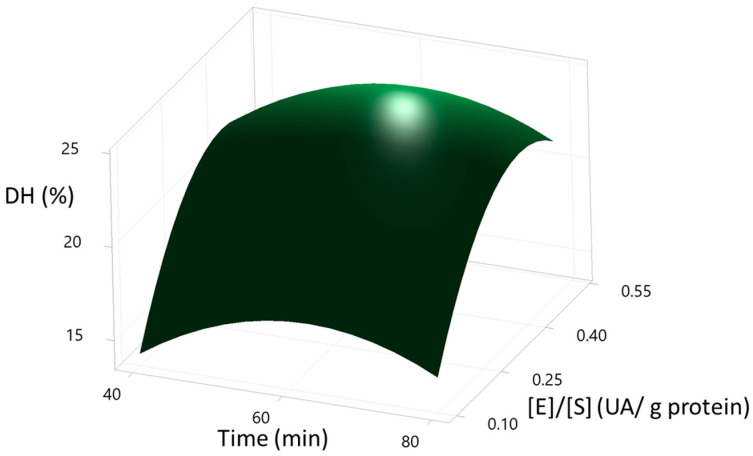
Surface plot of the degree of hydrolysis (DH%) of SVM sample vs. [E]/[S] (UA/g protein), time (°C). Hold Value: Temperature (50 °C).

The shapes of the contour curves can be seen in Figure 4, Figure 5 and Figure 6. The shapes of the curves indicate the significance of the interactions between the variables studied (temperature, time, and enzyme/substrate ratio), from which it can be deduced that temperature affects the DH % much more than time. In contrast, the enzyme/substrate ratio has a more significant effect on the DH % than temperature. All plots showed flared contours following a second-order quadratic regression model.

### 3.5. Characterisation of Protein Hydrolysates

Table 7 shows the characterisation of the SPH_FD_ and DSPH_FD_ samples. The moisture content of the SPH_FD_ and DSPH_FD_ was 3.11% and 4.54%, respectively, and were similar to the values reported in previous investigations in different hydrolysates of marine by-products [34,35,36]. The protein content for the samples SPH_FD_ and DSPH_FD_ were 69.58% and 71.67%, respectively, lower than 80% of the protein content reported by Idowu et al. [35] for salmon by-product hydrolysates but close to the values reported by Nurdiani et al. [36]. Ash content was 11.08% and 9.85% for SPH_FD_, and DSPH_FD_, respectively. These values were higher than those reported by Nurdiani et al. [36] (5.40 and 6.56%) because the ash content of hydrolysates is affected by the addition of acids or bases used to fix the pH in the process. Furthermore, releasing minerals from the bones or aquaculture by-products could explain the high ash content. The DH % of the SPH_FD_ sample was 20.44%. Other authors have reported a wide range of DH %, between 7% and 50% [24,36].

### 3.6. Amino Acid Profile of Protein Hydrolysates

Table 8 shows the amino acid profile of the SPH_FD_ and DSPH_FD_ samples. Twenty types of standard amino acids were identified, and the nine essential amino acids were found. The amino acid composition of the samples was similar, and the predominant essential amino acids were aromatic amino acids Tyr (2.92%), Phe (3.20%) and Trp (0.82%); and sulphur amino acids Met (3.39%), and Cys (0.79. The results indicate that Val (3.29%) is the limiting amino acids due to their lower content compared to OMS/WHO patron (4.0%). On the other hand, the proportion of essential amino acids regarding total amino acids were between 35% and 36%, which was close to the value (32.6 and 34.6%) reported by Xu et al. [37]. Regarding no essential amino acids both samples reported higher amounts of Gly (10.60%), which was slightly lower to the content (15.11%) reported by Zhi et al. [38]. The bioactive properties of protein hydrolysates are dependent on the composition and sequence of their amino acids [16]. For instances, the presence of hydrophobic amino acids (Ile, Ala, and Pro) and basic amino acids (Lys) in the peptide sequences contribute to the high antioxidant capacity [39]. According to Hwang and Winkler-Moser [40], amino acids containing additional thiol, thioether, or amine groups, such as Arg, Cys, Lys, Met, and Trp, showed substantial antioxidant activities. The amino acids Tyr (2.92%), Phe (3.20%), Pro (4.13%), Ala (5.95%), His (1.74%), and Leu (6.53%), categorised as antioxidants [16] were slightly higher than those reported by Bui et al. [16]: Tyr (1.76%), Phe (1.55%), Pro (2.89%), Ala (4.76%), His (1.2%), and Leu (5.43%).

### 3.7. Protein Solubility Curve of Hydrolysates

**Figure 7:**
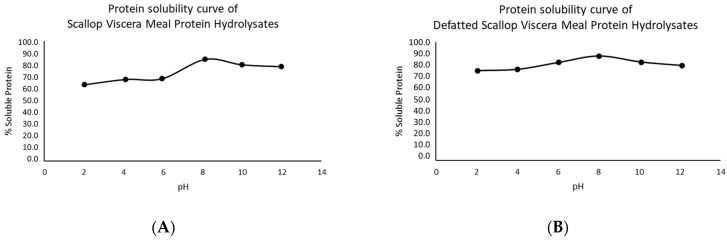
Protein solubility curve of freeze-dried protein hydrolysates from scallop viscera meal: (**A**) SPH_FD_ sample and (**B**) DSPH_FD_ sample.

The solubility curves of SPH_F_**_D_** and DSPH_FD_ samples are shown in Figure 7. The curve of the SPH_FD_ sample showed slightly lower values than the DSPH_F_**_D_** sample in the pH range of 2–6. This could be due to the deficiencies in forming emulsions for the fat content in the SVM sample. However, both hydrolysates have high solubilities (>60%), especially compared to the initial raw material, which barely exceeded 50% protein solubility. This behaviour was also presented by Mahdabi and Hosseini Shekarabi [32], where kilka fish hydrolysates (*Clupeonella* sp.) were more soluble than kilka fishmeal. According to Karami and Akbari-adergani [41], hydrolysates with smaller peptides, which mean a higher DH%, were more soluble. This coincides with the DH% of 20.44% for the SPH_FD_. On the other hand, the range that maximizes both hydrolysates were between 7 to 9, which was distinguish than the pH observed for SVM and SVMD, where the range were around 12. This could be explained due to hydrolysates has small molecular weight that are more polar [42], than the intact proteins of SVM and SVMD. Moreover, these differences could be due to the more electrostatic interactions of peptides in alkaline solutions than intact proteins. This could explain why SVMD and SVM needed higher pH than their hydrolysates to get maximum solubility.

### 3.8. Molecular Weight Profile of Protein Hydrolysates

**Figure 8:**
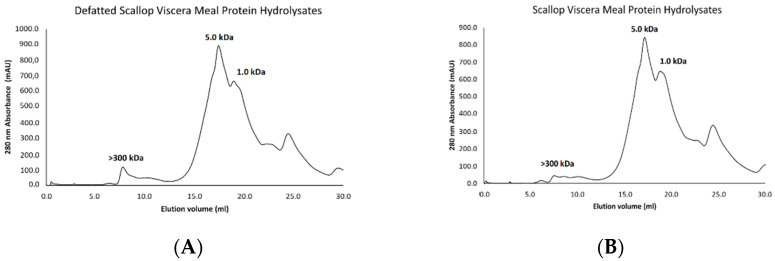
Molecular weight profile of the freeze-dried protein hydrolysates from scallop viscera meal evaluated in the Superose 12 column in a separation range between 300–1 kDa: (**A**) DSPH_FD_ sample; (**B**) SPH_FD_ sample.

The molecular weight of the hydrolysed proteins of scallop viscera flour is presented in Figure 8. Two almost identical chromatograms can be seen where most fractions have MW less than or equal to 5 and 1 kDa for SPH_FD_ and DSPH_FD_. This could be explained due to the specificity of the hydrolysis enzymatic. In fact, Gao et al. [7] mentioned that other method as acid or basic hydrolysis are no suitable to control de molecular weight. Several research shown that the molecular weight is related with some bioactive properties. As mentioned above, the bioactive properties of the hydrolysates depend on their composition and sequence and, therefore, on the average molecular weight [24]. Furthermore, peptides with low molecular weight, between 2–20 amino acids, showed highly efficacious bioactive properties than larger parent polypeptides/proteins [43]. Bui et al. [16] mentioned that bioactive peptides have a molecular weight of less than 50 kDa. Gao et al. [14] shown that lower the molecular weight, higher the antioxidant activity of the hydrolysate of Sturgeon muscles. On the other hand, the relation of the DH% regarding the molecular weight has been widely discussed. Rezvankhah et al. [44] proved that higher DH%, lower molecular weight in protein hydrolysate. According to Silvestre et al. [45] high-grade hydrolysis (20–50%) presents molecular weitght < 3 kDa, which is consistent with the results of this studio.

## 4. Conclusions

The protein hydrolysates were optimised using the response surface methodology, obtaining a predicted DH % of 24.99%. Both the scallop viscera flours and hydrolysed proteins presented balanced amino acid profiles according to the requirements established by the FAO/WHO. The molecular weight profiles were between 1–5 kDa, suggesting that the hydrolysates’ peptides could exhibit bioactive activities. This study could be taken into consideration as a starting point for further research of the bioactive properties of the hydrolysates such as antioxidant activity, antihypertensive activity, or anti-inflammatory activity.

## Figures and Tables

**Table 1:** Independent variables and coded values in Box-Behnken design for single-factor experiments.

Independent Variables	Coded Values		
	−1	0	+1
Temperature (°C) (X1)	30	50	70
Time (min) (X2)	40	60	80
Enzyme/substrate level (AU/g protein) (X3)	0.1	0.3	0.5

**Table 2:** Box-Behnken design to optimised the degree of hydrolysis, DH%.

Std Order	Run Order	Pt Type	Blocks	Temperature (°C)	Time (min)	[E]/[S] (AU/g de Protein)	Degree of Hydrolysis (DH%)
6	1	2	1	70	60	0.1	15.79 ± 0.07
11	2	2	1	50	40	0.5	18.19 ± 0.09
5	3	2	1	30	60	0.1	9.88 ± 0.21
3	4	2	1	30	80	0.3	14.08 ± 0.19
12	5	2	1	50	80	0.5	21.69 ± 0.29
4	6	2	1	70	80	0.3	21.76 ± 0.09
8	7	2	1	70	60	0.5	21.93 ± 0.2
14	8	0	1	50	60	0.3	23.78 ± 0.29
10	9	2	1	50	80	0.1	17.48 ± 0.12
13	10	0	1	50	60	0.3	23.63 ± 0.12
15	11	0	1	50	60	0.3	24.24 ± 0.15
1	12	2	1	30	40	0.3	17.34 ± 0.21
9	13	2	1	50	40	0.1	14.11 ± 0.15
2	14	2	1	70	40	0.3	20.3 ± 0.35
7	15	2	1	30	60	0.5	20.76 ± 0.05

Results are expressed as mean (*n* = 3) ± standard deviation (SD).

**Table 3:** Proximal composition of scallop viscera meal (SVM and SVMD).

Determination	SVM	SVMD
Moisture (%)	1.75 ± 0.17	7.03 ± 0.08
Protein (N × 6.25) (%)	72.23 ± 0.13	73.33 ± 0.95
Fat (%)	7.54 ± 0.14	nr
Ash (%)	9.54 ± 0.13	9.29 ± 0.97
Fibre (%)	6.75 ± 0.37	7.25 ± 0.37

Results are expressed as mean ± SD (*n* = 3). nr: No reported.

**Table 4:** Amino acid profile of scallop viscera meal (SVM and SVMD).

Amino Acids	SVM		SVMD		FAO % (mg Amino Acid/mg Total of Amino Acids)
	mg Amino Acid/g Protein	% (mg Amino Acid/mg Total of Amino Acids)	mg Amino Acid/g Protein	% (mg Amino Acid/mg Total of Amino Acids)	
Asp + Asn	66.57 ± 3.30	10.38 ± 0.55	47.74 ± 5.86	8.55 ± 1.31	
Glu + Gln	91.56 ± 2.59	14.27 ± 0.40	77.91 ± 3.63	13.92 ± 1.08	
Ser	34.85 ± 1.94	5.42 ± 0.18	38.91 ± 3.00	6.92 ± 0.32	
His	12.22 ± 0.82	1.90 ± 0.07	11.09 ± 1.31	1.97 ± 0.17	1.6
Gly	80.06 ± 5.72	12.46 ± 0.59	86.25 ± 8.69	15.33 ± 1.08	
Thr	32.45 ± 1.64	5.05 ± 0.14	29.18 ± 2.33	5.19 ± 0.25	2.5
Arg	59.70 ± 3.92	9.29 ± 0.39	56.96 ± 5.27	10.66 ± 0.61	
Ala	35.45 ± 0.97	5.53 ± 0.17	34.61 ± 1.23	6.18 ± 0.41	
Pro	5.49 ± 2.91	0.85 ± 0.43	9.19 ± 2.42	1.65 ± 0.48	
Tyr ^b^	20.33 ± 1.11	3.16 ± 0.07	20.07 ± 2.08	3.57 ± 0.26	
Val	35.89 ± 1.47	5.59 ± 0.06	10.65 ± 0.09	1.90 ± 0.04	4.0
Met ^a^	18.35 ± 0.72	2.86 ± 0.14	22.32 ± 2.31	3.79 ± 0.30	2.3 ^a^
Cys ^a^	6.83 ± 0.76	1.07 ± 0.14	11.12 ± 1.13	1.98 ± 0.14	
Ile	25.65 ± 1.16	3.99 ± 0.09	12.80 ± 0.47	2.28 ± 0.01	3.0
Trp	4.66 ± 0.00	0.73 ± 0.03	3.96 ± 0.00	0.71 ± 0.02	0.7
Leu	46.63 ± 2.16	7.26 ± 0.16	38.23 ± 1.55	6.81 ± 0.07	6.1
Phe ^b^	30.30 ± 1.75	4.72 ± 0.19	20.52 ± 2.44	3.65 ± 0.32	4.1 ^b^
Lys	35.04 ± 1.31	5.46 ± 0.04	27.76 ± 0.22	4.95 ± 0.19	4.8

Results are expressed as mean ± SD (*n* = 3). Note: According to the Food and Agriculture Organisation, ‘Assessment of the quality of dietary proteins in human nutrition’ (2013). ^a^: Met + Cys; ^b^: Phe + Tyr.

**Table 5:** Coefficients table of the Box-Behnken design obtained from the Minitab 19 software.

Term	Coef	Se Coef	T-Value	*p*-Value	VIF
Constant	23.881	0.448	53.28	0.000	nr
Temperature (°C)	2.215	0.274	8.07	0.000	1.00
Time (min)	0.633	0.274	2.31	0.027	1.00
[E]/[S] (AU/g protein)	3.164	0.274	11.53	0.000	1.00
Temperature (°C) × Temperature (°C)	−3.144	0.404	−7.78	0.000	1.01
Time (min) × Time (min)	−2.368	0.404	−5.86	0.000	1.01
[E]/[S] (AU/g protein) × [E]/[S] (AU/g protein)	−3.647	0.404	−9.03	0.000	1.01
Temperature (°C) × Time (min)	1.180	0.388	3.04	0.004	1.00
Temperature (°C) × [E]/[S] (UA/g protein)	−1.184	0.388	−3.05	0.004	1.00
Time (min) × [E]/[S] (UA/g protein)	0.034	0.388	0.09	0.930	1.00

Coe: codified coefficients, Se Coef: standard error of Coefficients, VIF: variance inflation factor, nr: No reported.

**Table 6:** Analysis of variance of the Box-Behnken design obtained from the Minitab 19 software.

Source	DF	Adj SS	Adj MS	F-Value	*p*-Value
Model	9	679.585	75.509	41.76	0.000
Linear	3	367.629	122.543	67.77	0.000
Temperature (°C)	1	117.741	117.741	65.11	0.000
Time (min)	1	9.615	9.615	5.32	0.027
[E]/[S] (UA/g protein)	1	240.272	240.272	132.88	0.000
Square	3	278.412	92.804	51.32	0.000
Temperature (°C) × Temperature (°C)	1	109.502	109.502	60.56	0.000
Time (min) × Time (min)	1	62.116	62.116	34.35	0.000
[E]/[S] (UA/g protein) × [E]/[S] (UA/g protein)	1	147.328	147.328	81.48	0.000
2-Way Interaction	3	33.544	11.181	6.18	0.002
Temperature (°C) × Time (min)	1	16.715	16.715	9.24	0.004
Temperature (°C) × [E]/[S] (UA/g protein)	1	16.816	16.816	9.30	0.004
Time (min) × [E]/[S] (UA/g protein)	1	0.014	0.014	0.01	0.930
Error	35	63.288	1.808		
Lack-of-Fit	3	61.571	20.524	382.55	0.000
Pure Error	32	1.717	0.054		
Total	44	742.873			

DF: degrees of freedom, Adj SS: adjusted sums of squares, Adj MS: adjusted sum of squares for the model.

**Table 7:** Characterisation of protein hydrolysates from scallop viscera meal.

Determination	SPH_FD_	DSPH_FD_
Moisture (%)	3.11 ± 0.44	4.54 ± 0.24
Ash (%)	11.08 ± 0.42	9.85 ± 0.25
Protein (Nx6.25) (%)	69.58 ± 0.36	71.67 ± 0.36
Yield	93.92	91.29
Colour	Dark browm	Dark browm
Degree of hydrolysis (DH %)	20.44 ± 1.02	-

Results are expressed as mean ± SD (*n* = 3).

**Table 8:** Amino acid profile of protein hydrolysates from scallop viscera meals.

Amin Acid	SPH_FD_		DSPH_FD_		FAO % (mg Amino Acid/mg Total of Amino Acids)
	mg Amino Acid/g Protein	% (mg Amino Acid/mg Total of Amino Acids)	mg Amino Acid/g Protein	% (mg Amino Acid/mg Total of Amino Acids)	
Asp + Asn	94.43 ± 0.88	13.68 ± 0.12	97.84 ± 9.03	12.74 ± 0.72	
Glu + Gln	110.18 ± 3.22	15.92 ± 0.36	117.03 ± 1.61	15.29 ± 0.40	
Ser	35.24 ± 0.20	5.09 ± 0.03	38.38 ± 1.24	5.01 ± 0.04	
His	12.05 ± 0.05	1.74 ± 0.01	13.32 ± 0.23	1.74 ± 0.04	1.6
Gly	73.32 ± 0.81	10.60 ± 0.04	79.69 ± 0.60	10.42 ± 0.50	
Thr	31.89 ± 0.45	4.61 ± 0.03	34.15 ± 0.23	4.47 ± 0.19	2.5
Arg	57.83 ± 1.26	8.36 ± 0.13	62.82 ± 0.51	8.22 ± 0.41	
Ala	41.15 ± 0.44	5.95 ± 0.02	44.70 ± 0.35	5.84 ± 0.20	
Pro	28.45 ± 0.41	4.13 ± 0.01	56.06 ± 11.23	7.08 ± 1.31	
Tyr ^b^	20.18 ± 0.47	2.92 ± 0.05	21.91 ± 0.29	2.87 ± 0.16	
Val	22.75 ± 0.52	3.29 ± 0.05	23.63 ± 0.21	3.09 ± 0.14	4.0
Met ^a^	23.44 ± 0.57	3.39 ± 0.05	24.71 ± 0.44	3.23 ± 0.16	2.3 ^a^
Cys ^a^	5.48± 0.05	0.79± 0.00	6.24 ± 0.17	0.81 ± 0.01	
Ile	26.24 ± 0.70	3.79 ± 0.06	28.25 ± 0.55	3.70 ± 0.23	3.0
Trp	5.70 ± 0.00	0.82 ± 0.01	5.77 ± 0.00	0.76 ± 0.03	0.7
Leu	45.20 ± 0.73	6.53 ± 0.06	49.04 ± 0.34	6.41 ± 0.30	6.1
Phe ^b^	22.14 ± 0.75	3.20 ± 0.09	23.94 ± 0.22	3.13 ± 0.16	4.1 ^b^
Lys	36.07 ± 0.63	5.21 ± 0.06	39.58 ± 0.32	5.18 ± 0.25	4.8

Results are expressed as mean ± SD (*n* = 3). Note: According to the Food and Agriculture Organisation, ‘Assessment of the quality of dietary proteins in human nutrition’ (2013). ^a^: Met + Cys; ^b^: Phe + Tyr.

## Data Availability

Not applicable.
